# Impact of music-based interventions on subjective well-being: a meta-analysis of listening, training, and therapy in clinical and nonclinical populations

**DOI:** 10.3389/fpsyg.2025.1608508

**Published:** 2025-07-09

**Authors:** Jie Zhang, Yanan Lu, Katayoun Mehdinezhadnouri, Junjie Liu, Haohui Lu

**Affiliations:** ^1^Faculty of Social Sciences and Liberal Arts, UCSI University, Kuala Lumpur, Malaysia; ^2^Department of Music, Zhengzhou University of Industrial Technology, Xinzheng, China; ^3^Department of Music, Faculty of Human Ecology, Universiti Putra Malaysia, Serdang, Selangor, Malaysia

**Keywords:** music-based interventions, subjective well-being, music listening, music training, music therapy

## Abstract

**Introduction:**

This meta-analysis aims to systematically evaluate the impacts of three types of Music-based interventions (MBIs)—music listening, music training, and music therapy on the subjective well-being (SWB) of clinical and non-clinical populations.

**Methods:**

The study conducted a systematic search of Web of Science, PubMed, and Scopus (from inception to January 2025) using the PRISMA guidelines, and selected 10 studies with a total of 387 and 326 experimental and control groups, respectively. Study quality was assessed using the Cochrane Risk of Bias Tool for randomized controlled trials. A random-effects meta-analysis was then performed in Stata 18.0 to compute standardized mean differences (SMDs) and 95% confidence intervals (CIs).

**Results:**

The pooled effect sizes indicated that MBIs were significantly associated with higher levels of SWB compared with control conditions (SMD = 0.36, 95% CI: 0.06–0.65, *p* = 0.02). Subgroup analyses revealed significant variations across intervention types and populations. Music listening was significantly associated with higher SWB in clinical groups (SMD = 0.65, 95% CI: 0.02–1.29); however, no significant association was found in nonclinical groups (SMD = 0.28, 95% CI: −0.14–0.70), although a positive overall association was observed (SMD = 0.42, 95% CI: 0.06–0.77). Music training showed a significant positive association with SWB in clinical groups (SMD = 1.76, 95% CI: 1.04–2.48), but no significant association was found in nonclinical groups (SMD = −0.32, 95% CI: −0.84–0.20) or in the overall sample (SMD = 0.00, 95% CI: −0.77–0.78). In contrast, music therapy was significantly associated with improvements in SWB across both clinical and nonclinical populations.

**Discussion:**

The results indicated that MBIs may improve SWB, though the strength of the association appears to vary depending on the type of intervention and the characteristics of the target population. Music training yielded the most significant effects in clinical populations, whereas music therapy was most effective in nonclinical populations. The effects of music training and music listening were less pronounced potentially due to differences in emotional needs, interactivity, and training difficulty. Future research should focus on individualized designs for intervention and further investigate the influence of factors such as type of intervention, duration, frequency, characteristics of participants, and reinforcement of interventions on the long-term effects on SWB.

**Systematic review registration:**

https://www.crd.york.ac.uk/prospero/, CRD42025641732.

## 1 Introduction

Music-based interventions (MBIs) employ structured approaches to influence psychological, physiological, and social outcomes through musical activities. In recent years, with the surge in psychological problems and illnesses, researchers have paid more attention to and attempted to use a variety of intervention measures. MBIs have received considerable attention and have achieved good results in alleviating people’s various negative emotions, such as anxiety, depression, and frustration (Rodwin et al., 2023), while also improving positive emotions, such as subjective well-being (SWB). SWB a construct that encompasses cognitive evaluations of life satisfaction and affective experiences of positive and negative emotions (Diener, 1984). Low levels of SWB are associated with increased risks of depression, anxiety, and impaired daily functioning, underscoring its importance as both a mental health indicator and a target for intervention in clinical and nonclinical populations [Diener et al., 2018; World Health Organization, 2024]. MBIs range from receptive listening to active participation, including playing instruments, singing, and structured music therapy, with varying levels of engagement and intensity (Burrai et al., 2016; Haslbeck et al., 2023; Schneider et al., 2022; Spintge, 2023). Empirical studies demonstrate that MBIs yield positive outcomes in clinical and nonclinical populations by enhancing emotion regulation, fostering positive affect, and improving overall well-being (Agres et al., 2021; Bainbridge et al., 2021; Juslin and Västfjäll, 2008; Kemper and Danhauer, 2005; Landis-Shack et al., 2017; Thaut and Hoemberg, 2014).

MBIs are typically categorized into active and receptive approaches. Which differ not only in the level of participation they require but also in the psychological and neural mechanisms they engage. Active MBIs, such as playing instruments, singing, or improvisation, involve direct physical and emotional engagement (Rodwin et al., 2023). These modalities are believed to promote emotional expression, social connectedness, and sensorimotor integration, and may activate subcortical brain structures associated with bottom-up emotion regulation (Clift et al., 2010; McFerran et al., 2020; Rodwin et al., 2023). In contrast, receptive MBIs, most commonly involving music listening or lyric analysis, tend to support top-down processes, including reflection, affective modulation, and cognitive appraisal. These approaches are widely used to reduce stress and enhance well-being by stimulating reward-related neural pathways and eliciting pleasurable emotional states (Feneberg et al., 2020; Koehler et al., 2023; Menon and Levitin, 2005). Beyond these modes, professional music therapy delivered by credentialed music therapists integrates both active and receptive techniques within a structured therapeutic framework (Hanser et al., 2006). Grounded in psychological and clinical principles, music therapy has demonstrated significant therapeutic benefits across diverse mental health conditions, particularly in clinical populations (de Witte et al., 2022). This multidimensional model underscores the importance of distinguishing between intervention types when evaluating the effectiveness of MBIs.

The mechanisms through which MBIs contribute to the enhancement of SWB are multifaceted and supported by empirical research. In terms of emotional regulation, music facilitates the identification, expression, and regulation of emotion. Previous studies indicate that listening to music reduces cortisol levels, which attenuates stress responses and promotes well-being (Jäncke, 2008; Juslin and Västfjäll, 2008; Koelsch et al., 2016). During neurophysiological processes, music activates neural circuits linked to reward processing and affective regulation, thus promoting the release of oxytocin and serotonin—neurotransmitters that are crucial for emotional stability and SWB (Chanda and Levitin, 2013). Another mechanism includes that of social connection and interpersonal bonding in which participation in group-based musical activities, such as choral singing or ensemble performances, strengthens social cohesion, decreases perceived loneliness, and enhances interpersonal relationships, which are key determinants of SWB (Galinha et al., 2022; Hanna-Pladdy and MacKay, 2011). The last pertains to the induction of flow states. Engagement in musical activities frequently induces flow states, characterized by deep concentration, intrinsic motivation, and an altered perception of time. Empirical findings indicate that experiencing flow during musical engagement is positively associated with increased well-being and life satisfaction (Loepthien and Leipold, 2022). It can be seen that MBIs have a certain degree of influence on positive emotions, negative emotions, and life satisfaction, which constitute SWB. Therefore, MBIs may become an effective method for improving SWB.

Although a substantial body of research supports the positive role of MBIs in elevating SWB, existing literature continues to present inconsistent. For example, Che et al. (2022) reported no significant improvements in SWB among nonclinical participants after listening to music or undergoing training, which was attributed to insufficient engagement and variations in music preferences. Similarly, Galinha et al. (2022) observed nonsignificant effects of music training on life satisfaction and negative emotions among nonclinical populations, underscoring the potential influence of individual differences and stability of psychological constructs. These findings emphasized the importance of tailoring interventions according to participants’ characteristics, preferences, and needs. Previous meta-analyses primarily focused on clinical outcomes, such as depression, anxiety, and stress reduction (Gold et al., 2009; Aalbers et al., 2019; Agres et al., 2021; Kim and Kang, 2021; Chen et al., 2022; McCrary et al., 2022), but have offered only limited insight into the broader impact of MBIs on well-being as defined by SWB. For instance, Gustavson et al. (2021) identified methodological limitations and highlighted inconsistent evidence on general mental health associations; Chen et al. (2022) emphasized the need for a neurocircuitry-informed framework; McCrary et al. (2022) demonstrated meaningful improvements in health-related quality of life across diverse MBI types. However, none explicitly examined SWB, nor did they disentangle effects by intervention modality or population type. This gap led to uncertainty regarding which types of MBIs are most effective for enhancing SWB in varying contexts. In response, our meta-analysis specifically examines how different MBIs (listening, training, therapy) affect SWB, and whether these effects differ between clinical and nonclinical adults, thereby addressing a clear need for enhanced specificity in intervention recommendations.

As MBIs are increasingly implemented across both clinical and community contexts, this study incorporates adult participants from both populations to assess whether intervention effects vary based on health status. While the inclusion of diverse populations introduces heterogeneity, it mirrors real-world practice and enables subgroup analyses to explore differential patterns of effectiveness. It is acknowledged that developmental factors, such as age, may shape how individuals perceive and report SWB. This potential variability is considered a limitation when interpreting pooled results across diverse adult populations.

The current study conducts a systematic review and meta-analysis to quantitatively evaluate the effects of MBIs on SWB. Specifically, it aims to (1) determine the overall impacts of MBIs on SWB, (2) compare the differential effects of various intervention modalities (e.g., music listening, training, and therapy), and (3) assess variations in these effects between clinical and nonclinical populations. By elucidating the effectiveness of different intervention strategies for distinct population groups, it intends to provide critical insights for optimizing the application of MBIs to research and practice.

## 2 Methods

The systematic review and meta-analysis were conducted in accordance with the Preferred Reporting Items for Systematic Reviews and Meta-Analyses 2020 statement (Page et al., 2021). The protocol was registered with the International Prospective Register of Systematic Reviews (registration number: CRD42025641732).

### 2.1 Search strategy

The study conducted a search of articles on the electronic databases of Web of Science, Scopus, and PubMed from their inception to January 15, 2025. These databases were selected for their broad and interdisciplinary coverage of health, psychological, and social science research, and have been widely used in prior systematic reviews on music-based interventions and well-being. The following search strategy was adapted for each database and combined under Boolean language: “Subjective Well-Being” OR “Emotional Well-Being” OR “Life Satisfaction” OR “Happiness” OR “Positive Affect” AND “Music Intervention” OR “Music Therapy” OR “Music Training” OR “Music Listening” OR “Music Performance.” AND “adult” OR “clinical” OR “non-clinical” The search strategy was developed by the authors in consultation with an expert in systematic review methodology in psychology and health sciences. The Supplementary material presents the detailed search strategy for each database. After removing duplicates, the researchers screened the title and abstract of each article for potential inclusion. In addition to database searches, we identified additional studies by screening reference lists of included articles and conducting targeted searches on academic platforms (e.g., Google Scholar, ResearchGate, institutional repositories). These studies were evaluated using the same inclusion criteria and quality assessment procedures. Four such articles were included in the final analysis.

### 2.2 Eligibility criteria

In accordance with the PICO framework (Page et al., 2021), studies were selected if they met the following criteria:

(1) Population: Participants were adults aged 18 years or older. Based on study-reported characteristics, samples were classified as clinical populations (e.g., individuals with diagnosed physical or mental health conditions) or non-clinical populations (e.g., community-dwelling adults without known diagnoses).
(2) Intervention: the studies examined the effects of a music intervention, which encompassed but was not limited to music listening, performance, singing, or therapy.
(3) Comparator: the studies included a control group that received no music intervention or was subjected to an alternative nonmusic intervention (e.g., physical exercise, psychological intervention, or no intervention).
(4) Outcomes: SWB was assessed using at least one of the following validated instruments: Satisfaction with Life Scale (Diener et al., 1985), Positive and Negative Affect Schedule (Watson and Levin-Aspenson, 2018), Basler Mood Questionnaire (Myrtek, 2004), General Well-Being Scale (Longo et al., 2017), or Warwick–Edinburgh Mental Well-Being Scale (Tennant et al., 2007), among others. These measures were selected based on widely accepted conceptualizations of SWB as comprising both cognitive and affective components (Diener et al., 1985), and their common use in prior systematic reviews on well-being outcomes in music-based interventions.
(5) Study design: studies that employed randomized controlled trials, quasi-experimental designs, or cross-sectional studies, including intervention and retrospective studies that incorporated randomized control groups.
(6) Publication Type: only peer-reviewed articles published in English were considered.

Studies were excluded based on the following criteria:

(1) Population: Included only participants under 18 years of age.
(2) Intervention: Focused solely on music education or cognitive training without a therapeutic or well-being-related objective.
(3) Outcomes: Did not assess SWB using validated instruments. Assessed only related constructs (e.g., anxiety, depression, stress, or cognition) without a direct measure of well-being.
(4) Study design: Lacked an intervention or control group
(5) Data availability: Reported results only in graphical form without accessible numerical data, and authors could not be reached for clarification.
(6) Publication type: Not peer-reviewed (e.g., conference proceedings, dissertations, or preprints). Not published in English.

### 2.3 Study selection and data extraction

All records were imported into Endnote X9 (Clarivate Analytics, Philadelphia, PA, USA), and duplicates were removed by the first author. Title and abstract screening was independently conducted by the first, second, and third authors using predefined eligibility criteria. To minimize bias, this process was performed in a blinded manner. Full-text screening was conducted using a standardized form, and discrepancies were resolved through discussion. The fourth and fifth authors contributed to resolving disagreements when consensus could not be reached. Inter-rater agreement was assessed at both title/abstract and full-text screening stages.

Data extraction was led and verified by the first author using a structured Excel spreadsheet. The second and third authors independently reviewed the extracted data for accuracy. Extracted items included publication details (author, year, journal), participant characteristics (e.g., age, sex, sample size), type of intervention (e.g., music therapy, listening, training), comparator group, outcome measures related to SWB, and intervention features (e.g., duration, frequency, delivery format, clinical status). To ensure standardized and transparent reporting of music-based interventions, intervention components were coded based on Robb et al. (2011) reporting guidelines. These included delivery mode (individual vs. group), facilitator qualifications, session frequency and length, music selection method (e.g., participant-selected vs. predefined), and stated therapeutic goals. The fourth and fifth authors supported data checking.

Quantitative data, including means and standard deviations, were directly extracted from the text. In the case of missing essential information, then the authors of the studies were contacted via email for clarification. If data were reported solely in graphical format, and the authors did not provide numerical data upon request, WebPlotDigitizer software (version 4.5) was utilized for data extraction. When reported, standard errors were subsequently converted into standard deviations *post hoc*. Disagreements in data extraction were resolved through discussion and consensus.

### 2.4 Assessment of study quality

The first and second authors independently evaluated the quality of each study using Cochrane Collaboration (Higgins and Green, 2008). Discrepancies in assessments were resolved by discussing with or seeking input from the third and fifth authors.

### 2.5 Statistical analysis

A random-effect meta-analysis was conducted for each outcome using Stata 18.0 (Stata Corp, TX, USA). In accordance with established guidelines for effect size computation for pre-and posttest intervention studies (Morris, 2008), the mean difference for each group was calculated as M_post − M_pre with the pretest standard deviation (SD_pre) used for standardization. Given the variability in outcome measures across studies, standardized mean differences (SMDs) were computed using Cohen’s *d* along with 95% confidence intervals (CIs). The interpretation of SMD values followed Cohen’s classification (Cohen, 1988): 0.2–0.49, 0.5–0.79, and >0.8 denoted small, moderate, and large effects, respectively.

Heterogeneity across studies was evaluated using *I*^2^ in which thresholds of 25, 50, and 75% represented low, moderate, and high heterogeneity, respectively (Higgins et al., 2003). If multiple task measures were used for a given outcome, then the most frequently applied measure was selected for analysis. After the computation of overall effect sizes for each outcome, subgroup analyses were performed to examine the differential effects of various MBIs. Publication bias was assessed through funnel plot visualization, while statistical significance was evaluated using Egger’s test with 95% CIs.

## 3 Results

### 3.1 Search results

**Figure 1:**
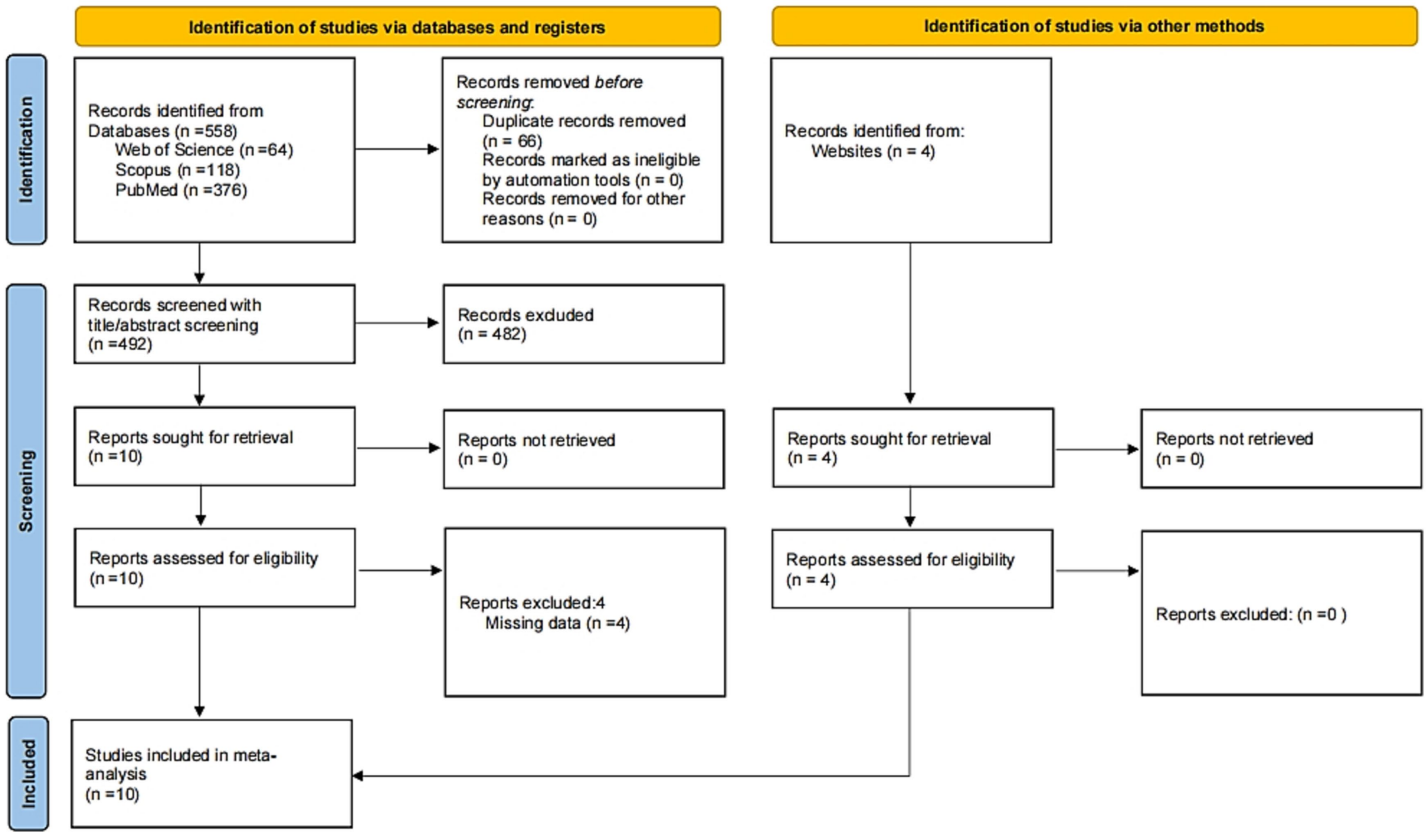
Document screening flow chart (adapted from PRISMA Statement; Page et al., 2021).

Figure 1 presents a flow diagram of the search and screening process. The initial search retrieved 558 articles, of which 492 articles remained after omitting duplications. The title and abstract screening excluded an additional 482 articles. Subsequently, 10 full-text articles were assessed for eligibility. Based on the inclusion criteria, 4 articles were deemed ineligible. Specifically, Fancourt et al. (2019) and Ji et al. (2023) did not provide complete data for the measurement of SWB. Lepping et al. (2016) did not conduct pre-and posttests, while Lund et al. (2020) only developed a study design without providing data. The remaining four articles, identified through websites, underwent the same rigorous quality assessment as those retrieved from database searches. Consequently, 10 studies were included for quantitative synthesis.

### 3.2 Study characteristics

The intervention details of the included studies including type, frequency, duration, setting, assessment task, and provider qualifications—were summarized in accordance with the reporting standards for music-based interventions proposed by Robb et al. (2011) and are presented in detail in Table 1. Outlines the characteristics of the 10 selected articles, which represent diverse regions (China (Shan and Qi, 2024), the United States (Innes et al., 2018), India (Gupta and Gupta, 2015), Spain (Castillejos and Godoy-Izquierdo, 2021; de la Torre-Luque et al., 2017), Germany (Bieligmeyer et al., 2018), Portugal (Galinha et al., 2022), the United Kingdom (Fancourt et al., 2016; Hanser et al., 2006; Che et al., 2022)). A total of 713 participants were included with 387 and 326 assigned to experimental and control groups, respectively. Notably, Hanser et al. (2006) examined patients with breast cancer, while Gupta and Gupta (2015) focused on male patients with coronary heart diseases. Gender differences in disease manifestation and specific clinical contexts drive this selective focus on particular genders in research design with the objective of gaining an in-depth understanding of the potential effects of MBIs under various physiological and psychological conditions.

**Table 1:** Basic characteristics of the selected studies.

Study	Region	Age (years)	Subjects (E/C)	Frequency	Duration	Experimental group	Control group	Assessment task	Qualifications
Shan and Qi (2024)	Shanghai, China	≥18 years	E: 102/C: 90	60 min/session, once per week	2 weeks	Music therapy under the PERMA framework Individualized music therapy plans Music priming sessions Musical expression activities Group music therapy to enhance social interaction Music relaxation (20 min per day) Regular assessments and adjustments	Routine nursing care (no specific music therapy)	General Well-Being Scale	Trained healthcare professionals and music therapists implementing the PERMA framework
Gupta and Gupta (2015)	Varanasi, India	43–64 years	E: 40/C: 40	30 min/day	20 days	Music subgroup listened to slow-paced instrumental music (ra-ga Desi-Todi) for 30 min per day for 20 days	Control subgroup did not receive music intervention; they sat in silence for 30 min daily for 20 days	Satisfaction with Life Scale	Music intervention was led by trained assistants. The music used was a slow-paced flute recording of ra-ga Desi-Todi
Castillejos and Godoy-Izquierdo (2021)	Spain	67–100 (mean: 84.82)	E: 25/C: 25	45–50 min/session. 2 times/week	6 weeks	12 group sessions (45–50 min each) and 2 individual sessions (30–45 min each) Sessions included listening to preferred music, singing, dancing, and playing instruments Music was selected based on participants’ preferences and designed to enhance physiological and mental states	Participants received standard nursing home care and were placed on a waiting list for music intervention	Affective Balance Scale Life Satisfaction (PANAS)	Psychologist trained and qualified in designing and delivering music interventions
Fancourt et al. (2016)	United Kingdom (London)	Mean: 55.07 (SD: 13.0) for drumming group; mean: 52.00 (SD: 14.7) for control group	E: 30/C: 15	90 min/session 1 times/week	10 weeks	Weekly 90-min sessions led by a professional drummer with experience in community music activities Sessions included basic drumming techniques, call-and-response exercises, and rhythmic patterns Participants used djembe drums and sat in a circle	Engaged in regular community-based social activities (e.g., quiz nights, book clubs) but did not participate in any group musical activities	Warwick–Edinburgh Mental Well-being Scale	Professional drummer with experience in leading community music activities
de la Torre-Luque et al. (2017)	Spain (Granada)	21.74 ± 3.26	E: 28/C: 30	15 min (one session)	Sep 2010–Feb 2011 single session	Participants listened to their preferred relaxing music for 15 min during the recovery period after stress exposure. Participants selected the music to induce relaxation	Participants rested silently for 15 min during the recovery period after stress exposure No music was played	Positive affect (PANAS) Negative affect (PANAS)	Research team with expertise in psychophysiology and music interventions
Innes et al. (2018)	West Virginia, USA	≥50	E: 11/C: 11	15–20 min/session. 2 times/day	8 weeks	Participants received a CD with 15-and 20-min tracks of instrumental music Practice involved listening to selected music for 15–20 min twice daily and recording sessions in a practice log	Participants received a CD with 15- and 20-min tracks Practice involved selecting a mantra, repeating it silently for 15–20 min twice daily	Profile of Mood States Psychological Well-Being Scale	A team member familiar with both programs and with experience in teaching relaxation techniques
Hanser et al. (2006)	Urban New England	Median: 53 (music training [MT] group); 50 (control)	E: 20/C: 22	45 min/session. 3 sessions	6 weeks (intervention period); follow-up at 6 weeks and 3 months	Three individual sessions (45 min each) Activities: live music, improvisation, songwriting, and relaxation techniques	Typical oncology and supportive care (no music therapy intervention)	Physical Well-Being Subscale Emotional Well-Being Subscale	Music therapists experienced in research; protocol designed by the first author (Suzanne Hanser)
Che et al. (2022)	Bath, UK	28–31 years old	E1: 10 E2: 11/ C: 10	60 min/session. once per week, 11 weeks	13 weeks (2 weeks after training ended)	MT received 11 weeks of piano training, which included finger exercises and learning piano songs using a Yamaha digital piano Music listening (ML) group listened to the same piano music used in the MT group for 1 h per week; no active music training, only receptive listening	Control (C) group: no musical input; spent training time doing regular homework; no music-related activities	Positive and Negative Affect Schedule (PANAS)	Music Trainer: the experimenter had 12 years of private piano education and held a certificate equivalent to ABRSM piano grade 8
Bieligmeyer et al. (2018)	Germany	Mean: 54.4 years	E: 21/C: 23	10 min/session 1 times/day	2 days	The experimental group received a soundbed music intervention based on the TAO pentatonic scale	Control patients were lying on sound beds but without music playing	Basler Mood Questionnaire	Soundbed performances by authors trained by music therapists with many years of soundbed experience
Galinha et al. (2022)	Portugal	Mean: 76.66 years	E: 89/C: 60	120 min/session 2 times/week	17 weeks	Participants engaged in a program called Sing4Health, which included relaxation and vocal warm-up exercises, vocal technique, repertoire rehearsal, a social component, and the creation and presentation of a final performance	The control group participated in regular social, arts, and leisure activities apart from group singing	Life Satisfaction (PANAS)	The intervention was carried out by professionally trained researchers who received specialized training as music therapists

The frequency of training sessions varied across studies with the majority of interventions conducted one to two times per week. However, Gupta and Gupta (2015) and Innes et al. (2018) reported daily interventions with the latter conducting sessions two times per day. Conversely, de la Torre-Luque et al. (2017) implemented one 15-min session. The duration of interventions ranged from 2 to 12 weeks with the exception of de la Torre-Luque et al. (2017), which consisted of only one session. Moreover, all studies assessed SWB.

### 3.3 Risk of bias assessment

**Figure 2:**
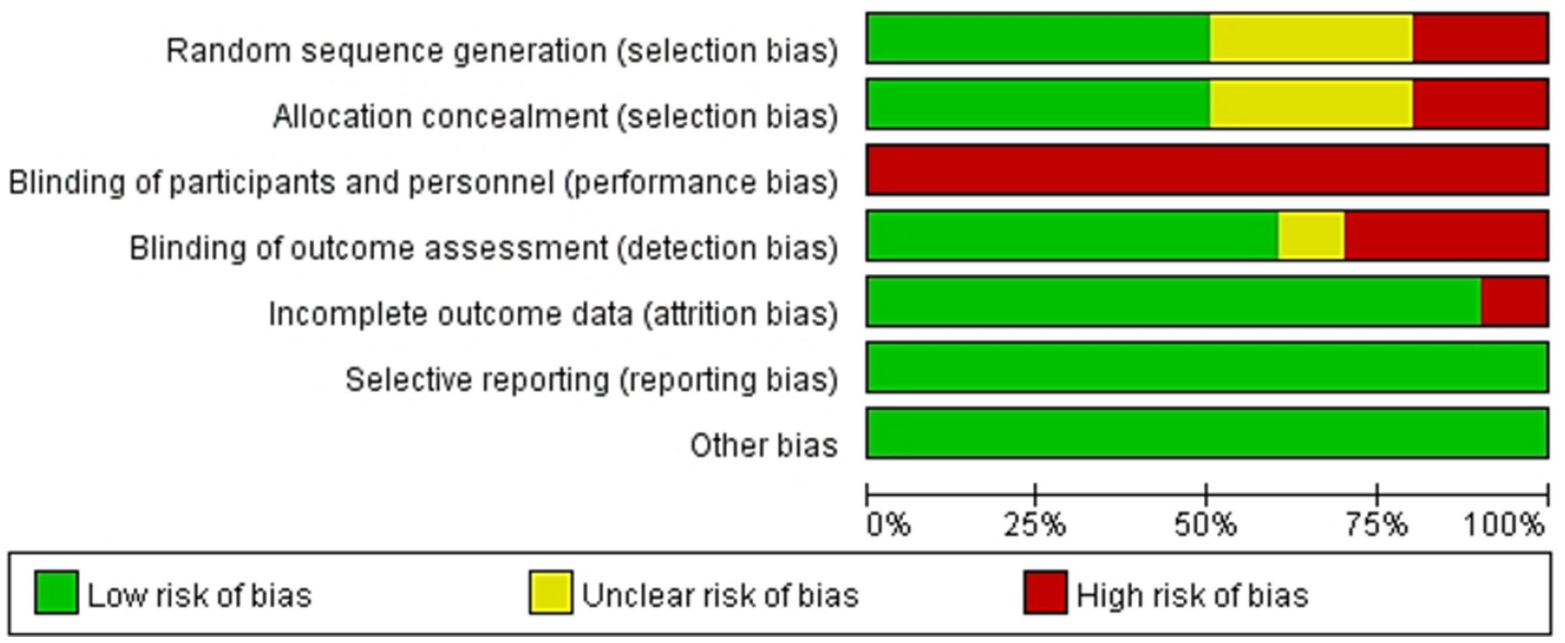
Risk of bias graph.

Figure 2 illustrates the risk of bias assessment. Five studies explicitly detailed the methods used to generate a randomized sequence, whereas three studies did not provide descriptions, and two studies did not perform randomized sequence generation. Five studies implemented centrally randomized allocation, whereas three studies did not describe the process of allocation concealment. Two studies have not been assigned concealment. Furthermore, blinding of participants was not feasible due to the inherent nature of the intervention, which resulted in a high risk of bias across the studies. Outcome measures were blinded in six studies, while nine studies reported complete outcome data. Furthermore, all studies exhibited low selectivity bias. No other biases were noted.

In training intervention studies, subjects were explicitly informed whether they are receiving an experimental intervention. This aspect can lead to performance bias in which knowledge of the intervention being given influences the behavior of subjects. In addition, the lack of detailed descriptions of random sequence generation and allocation concealment poses a significant risk for selection bias, which can affect the validity and reliability of study outcomes. Thus, addressing these issues in future research through a more rigorous methodological practice is essential for enhancing the robustness of findings in this field.

### 3.4 Meta-analysis

**Figure 3:**
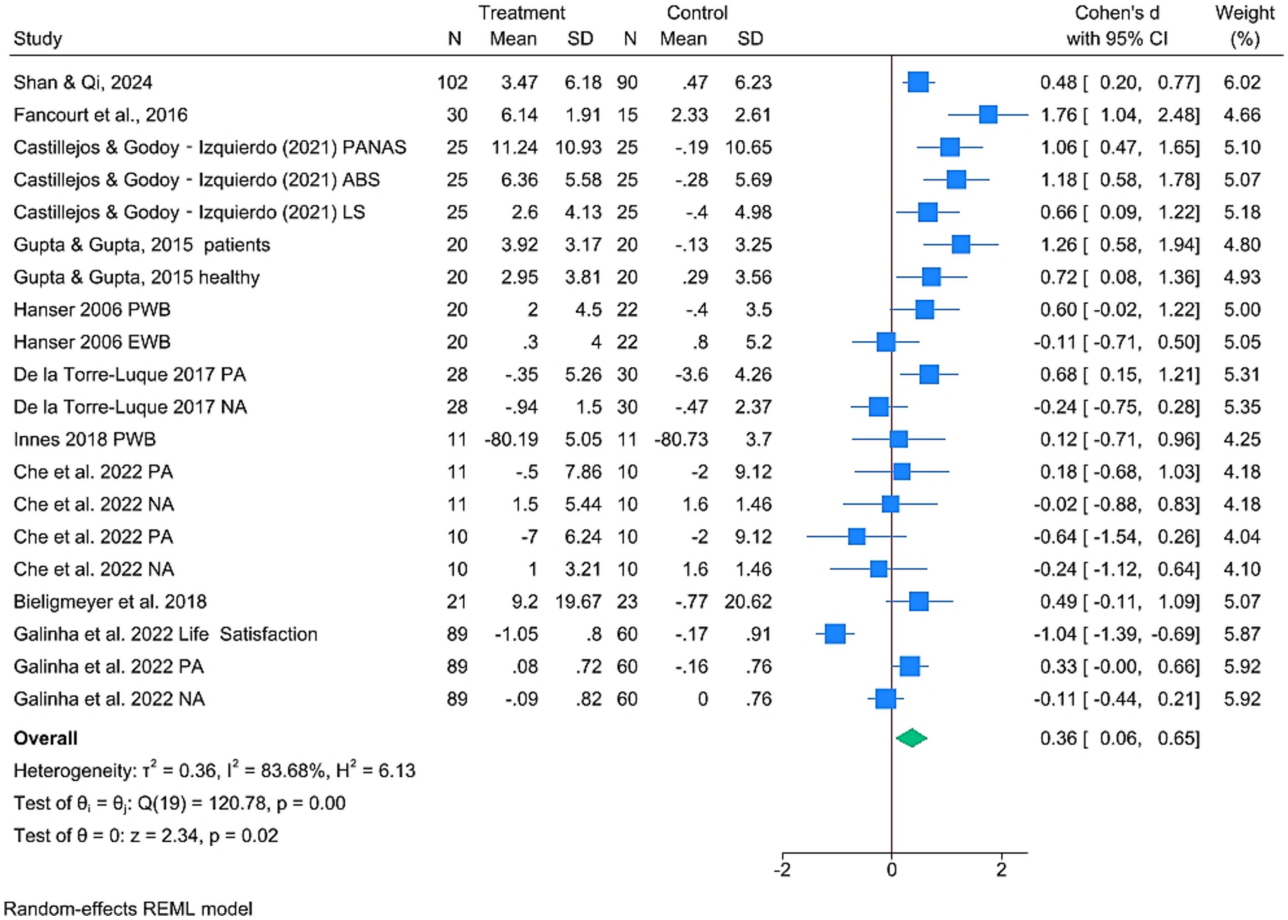
Forest plot of meta-analysis of SWB.

Ten studies examined the effects of MBIs on SWB, which yielded 20 effect sizes (Figure 3). Pooled analysis demonstrated a statistically significant association between MBIs and higher levels of SWB compared with control groups (SMD = 0.36, 95% CI: 0.06, 0.65, *p* = 0.02). However, substantial heterogeneity was observed (*I*^2^ = 83.68%, *p* < 0.001) potentially due to variations in the types of intervention (i.e., music listening, training, and therapy), participant demographics (e.g., gender, age, and health status), and measurement tools. Differences in scale sensitivity and specificity could have further contributed to these inconsistencies. Furthermore, Egger’s test revealed no significant publication bias (*p* = 0.6907, 95% CI: −2.54, 3.84). Subgroup analyses revealed (Table 2) that music listening (SMD = 0.42, 95% CI: 0.06–0.77, *p* = 0.02) and music therapy (SMD = 0.63, 95% CI: 0.29–0.97, *p* < 0.001) were significantly associated with improvements in SWB among adults, with music therapy showing a stronger association. For music listening, the clinical group showed a statistically significant improvement in SWB (SMD = 0.65, 95% CI [0.02, 1.29], *p* = 0.04, *I*^2^ = 59.67%), while the nonclinical group did not show a significant effect (SMD = 0.28, 95% CI [−0.14, 0.70], *p* = 0.19), although both subgroups exhibited moderate heterogeneity. In music training, a large and statistically significant effect was observed in the clinical group (SMD = 1.76, 95% CI [1.04, 2.48], *p* < 0.001), whereas the nonclinical group demonstrated a nonsignificant negative effect (SMD = −0.32, 95% CI [−0.84, 0.20], *p* = 0.23), with substantial heterogeneity (I^2^ = 84.62%). For music therapy, both groups benefited significantly from the intervention. The clinical group had a moderate effect (SMD = 0.37, 95% CI [0.02, 0.72], *p* = 0.04, *I*^2^ = 37.54%), while the nonclinical group showed a larger effect (SMD = 0.95, 95% CI [0.62, 1.29], *p* < 0.001), with no observed heterogeneity (*I*^2^ = 0%). These findings suggest that clinical populations may benefit more consistently from MBIs in terms of SWB, particularly in the context of music training and listening. Meanwhile, music therapy appears to be broadly effective across both clinical and nonclinical populations.

**Table 2:** Subgroup analysis of subjective well-being.

Outcome	Moderator	Subgroup	*N*	Heterogeneity test results			Meta-analysis results		
				Q	*p*	*I*^2^%	SMD	95% CI	*p*
Subjective well-being	Music listening	Clinical group	106	4.90	0.09	59.67%	0.65	[0.02, 1.29]	0.04
		Nonclinical group	119	8.40	0.08	51.81%	0.28	[−0.14, 0.70]	0.19
		Overall	225	15.76	0.03	55.00%	0.42	[0.06, 0.77]	0.02
	Music training	Clinical group	45	0.00			1.76	[1.04, 2.48]	0.00
		Nonclinical group	169	32.92	0.00	84.62%	−0.32	[−0.84, 0.20]	0.23
		Overall	214	61.54	0.00	93.72%	0.00	[−0.77, 0.78]	0.99
	Music therapy	Clinical group	234	3.41	0.18	37.54%	0.37	[0.02, 0.72]	0.04
		Nonclinical group	50	1.71	0.42	0.00%	0.95	[0.62, 1.29]	0.00
		Overall	284	11.76	0.04	60.27%	0.63	[0.29, 0.97]	0.00

## 4 Discussion

This systematic review and meta-analysis offer the first comprehensive evaluation of the effects of different types of MBIs (i.e., music listening, training, and therapy) on SWB across clinical and nonclinical populations. While the current results validate the positive impacts of MBIs on SWB, subgroup analyses reveal that factors such as intervention types, participant characteristics, and methodological variations influence these effects.

### 4.1 Music therapy

Music therapy significantly was associated with improvements in SWB. While the effect was significant in clinical groups, it was even more pronounced in nonclinical groups. From a psychological perspective, nonclinical populations are not affected by severe pathological conditions and primarily experience daily, transient stress. This context places them in a relatively advantageous position in terms of psychological resources (e.g., coping strategies and self-efficacy) and adaptability, rendering them more sensitive to emotional release and relaxation experiences in music therapy. Additionally, as this group did not undergo prolonged chronic psychological distress, they exhibit higher levels of psychological flexibility and sensitivity to positive emotional stimuli (Mac Donald et al., 2013). Consequently, they are more likely to experience immediate and intense emotional improvement and enhanced well-being during music therapy. At the physiological level, the neurophysiological systems of nonclinical populations remain unfazed by long-term pathological conditions, enabling heightened responsiveness to external stimuli such as music. Research demonstrates that music therapy may decrease cortisol levels and promote the release of neurotransmitters associated with positive emotions, by modulating the activity of the hypothalamic–pituitary–adrenal axis (McPherson et al., 2019).

The greater effectiveness of music therapy observed in this meta-analysis may be attributed to its unique interactive design (e.g., improvisation and lyrics analysis), which facilitates deep emotional resonance and social support. Conversely, music training primarily focuses on skill acquisition and does not provide the same level of emotional interaction (Knapik-Szweda, 2020). Furthermore, the literature demonstrates that music therapy may engage the reward pathways of the brain and mitigate stress responses (Chanda and Levitin, 2013), potentially eliciting more intense positive emotional experiences, particularly in nonclinical populations (Sambasivam et al., 2016).

Specifically, music therapy has been associated with improved emotional regulation through multiple mechanisms. For example, it facilitates emotion recognition through affective matching and lyric-based communication (Koelsch et al., 2010) and promotes emotional expression through rhythmic activities and improvisation (Fancourt et al., 2016). Furthermore, it regulates emotions by decreasing cortisol levels (Jäncke, 2008; Juslin and Västfjäll, 2008; Koelsch et al., 2016), enhancing neural plasticity, and providing self-regulation strategies (Chanda and Levitin, 2013). Taken together, these mechanisms may underlie the relatively stronger associations observed for music therapy in our results. These results align with those of previous findings indicating that structured and interactive music therapy effectively promotes emotional regulation and psychological well-being (Hanser et al., 2006).

Existing clinical research findings support the short-term efficacy of music therapy. For instance, Shan and Qi (2024) indicated that the music therapy group exhibited significantly decreased levels of anxiety and depression, along with notable improvements in SWB and quality of life, compared with the control group. However, in a study on patients with breast cancer, Hanser et al. (2006) reported that while relaxation and comfort significantly increased in the short term, follow-up assessments indicated that these effects were not sustained. The potential reasons underlying this result may be the brief duration of the intervention (three individual sessions of music therapy) and the lack of personalized design and emotional regulation training. Therefore, ongoing therapeutic support and individualized treatment may be key factors in maintaining the long-term effects of music therapy.

In a non-clinical population, the potential of music therapy in enhancing well-being and reducing stress, but also highlighted methodological limitations such as a short intervention period, insufficient follow-up, and limited intensity (Castillejos and Godoy-Izquierdo, 2021). Future efforts should optimize intervention design by extending the duration, increasing follow-up frequency, and tailoring programs to individual characteristics (such as age, personality, culture, and musical preferences) to enhance intervention effectiveness and sustainability. For example, older adults may benefit from music activities focused on emotional support, while younger adults may engage in more challenging creative activities.

### 4.2 Music listening

Compared with music therapy, the effects of music listening on different populations exhibit greater heterogeneity. In clinical populations, music listening significantly improves SWB, whereas this effect is nonsignificant in nonclinical populations. The positive effects of music listening on SWB can be further elucidated through the frameworks of emotion regulation theory (Gross, 2002) and the stress buffering model (Cohen and Wills, 1985). While such mechanisms are supported by neuroimaging literature (e.g., Chanda and Levitin, 2013), they were not directly assessed in the included studies and should be interpreted cautiously in the current context. However, its impact among nonclinical populations appears more variable, possibly due to greater individual differences in music preferences, engagement levels, and baseline emotional states (Gupta and Gupta, 2015; Che et al., 2022).

Clinical studies support these mechanisms by demonstrating short-term improvements in emotional states through music listening interventions. For instance, Innes et al. (2018) found that listening to music for 15–20 min twice daily across an eight-week period significantly decreased anxiety levels and enhanced positive emotions in patients with knee osteoarthritis. Bieligmeyer et al. (2018) achieved short-term improvements in mood among patients with cancer using a TAO pentatonic scale vibroacoustic music intervention. Notably, these interventions significantly vary in terms of type of music, mode of stimulation, and duration, while their long-term effects remain unclear.

In non-clinical populations, the effects of music intervention exhibit complexity. Gupta and Gupta (2015) found that music listening significantly improved life satisfaction, optimism, and hope in non-clinical individuals, consistent with results in clinical populations, demonstrating its positive effects. However, Che et al. (2022) found that in an 11-week piano music intervention, only positive emotions improved, while negative emotions showed no significant changes, possibly due to mismatches in individual music preferences and differences in participation levels. de la Torre-Luque et al. (2017) also reported that short-term (15-min) listening to self-selected relaxing music after acute stress only improved positive emotions, suggesting that insufficient intervention duration may limit effectiveness. Studies consistently highlight that individual differences (such as openness to experience, musical sensitivity, and baseline psychological state) and intervention design (duration, degree of personalization) are key factors influencing intervention outcomes.

Future studies should explore dose–response relationships by systematically examining intervention duration (e.g., short-vs. long-term), frequency (e.g., daily vs. weekly), and the degree of personalization (e.g., self-selected vs. researcher-selected music). Methodological limitations, such as small sample sizes, diverse intervention protocols, and reliance on self-reported measures, should be addressed. The use of objective assessments (e.g., physiological indicators, behavioral outcomes) and standardized protocols would help clarify the long-term effects of music listening on SWB. Additionally, greater attention to cultural contexts and preferences is needed to ensure broader applicability of findings.

### 4.3 Music training

Music training exhibited complex effects with significant discrepancies between clinical and nonclinical groups. The clinical groups demonstrated strong positive effects, whereas the nonclinical groups did not display significant improvements, which resulted in an overall negligible effect size. These differences may stem from variation in emotional needs, regulation capacities, and engagement levels between the two populations. Clinical populations typically experience significant psychological stress or emotional disorders, thus making them more likely to benefit from the opportunity for emotional expression, enhanced neuroplasticity, and social interaction offered by music training. In contrast, nonclinical populations demonstrated relatively lower levels of psychological needs, because they generally possess greater psychological resilience and stronger social support systems, which leaves a limited room for further improvement. This outcome may contribute to the presence of a *ceiling effect* in intervention outcomes.

The effectiveness of music intervention may be closely related to the “flow state” it induces. According to flow theory (Csikszentmihalyi and Csikzentmihaly, 1990), immersion, intrinsic motivation, and focus can enhance mood, reduce stress, and increase a sense of accomplishment. Group music activities are more likely to induce “group flow,” enhancing emotional resonance, a sense of belonging, and self-efficacy, thereby increasing well-being. Fancourt et al. (2016) found that structured group drumming significantly improved mental health in clinical populations. However, results in non-clinical populations are inconsistent, possibly due to intervention design. For example, Galinha et al. (2022) found that group singing improved positive emotions but did not enhance life satisfaction, possibly because performance pressure weakened the intervention effect; Che et al. (2022) found that one-on-one piano lessons did not significantly improve mood, possibly due to an overemphasis on technical skills and neglect of individual differences and emotional engagement. Therefore, future interventions should place greater emphasis on content appropriateness, taking into account participants’ abilities, motivations, and individual differences to maximize the positive impacts of music training.

Although the majority of the selected studies did not systematically explore gender factors, research that focused on specific conditions (e.g., breast cancer and coronary heart disease) indicates that gender may moderate the effects of MBIs in certain clinical contexts. For example, Hanser et al. (2006) reported that music therapy significantly enhanced SWB among female patients with breast cancer, which indicates that gender differences may pose important clinical implications in specific disease settings. However, subgroup analyses failed to reveal any universal differences based on gender, which may imply that the impact of gender on MBIs is not generalizable under non-disease-specific conditions. Future research should explore the influence of gender differences on responses to MBIs, because the results could inform the development of targeted and effective intervention strategies to meet the distinct needs of men and women.

### 4.4 Limitations and recommendations

Despite strong evidence that supports the positive effects of MBIs on SWB, certain pertinent limitations must be acknowledged. First, the high degree of heterogeneity among studies (*I*^2^ = 83.68%, *p* < 0.001) implies that differences in intervention protocols, participant demographics, and measurement tools may contribute to the inconsistencies in the findings. Additionally, several studies rely on self-reported measures of SWB, which are susceptible to social desirability bias and individual interpretation and potentially limits the objective assessment of the effects of MBIs (Cheng et al., 2022). Furthermore, the lack of standardized protocols for the implementation of MBIs constrains comparability across studies (Robb et al., 2011). Future research should incorporate objective measures, such as physiological assessments or neuroimaging, to complement self-report data. Moreover, the risk of publication bias remains a concern, as studies that present null or negative findings are less likely to be published (Song et al., 2010). Although Egger’s test indicated no significant publication bias (*p* = 0.6907, 95% CI: −2.54, 3.84), future meta-analyses could evaluate this risk and include preregistered studies to ensure a balanced representation of results. Variability within clinical populations—such as differences in diagnosis type and illness severity (e.g., cancer vs. cardiac conditions)—may have contributed to heterogeneity in outcomes. However, due to the limited number of studies per diagnostic group, subgroup analyses by condition were not feasible in the current review, highlighting an important area for future research. Although this review included three major interdisciplinary databases (Web of Science, Scopus, and PubMed), future studies may benefit from expanding the search to additional field-specific sources, such as PsycINFO or CINAHL, to improve coverage of psychological and healthcare literature. In addition, limiting the search to English-language publications may introduce language bias; future reviews should consider including non-English studies to capture a more comprehensive and globally representative evidence base.

Building on current findings, future research should focus on several key areas:

(1) Standardization of interventions: Developing clear guidelines regarding the duration, frequency, and music selection of interventions could improve comparability and replicability across studies.
(2) Mechanisms of action: Investigating the neurophysiological and psychological mechanisms underlying the impact of music on SWB could provide valuable insights into the optimization of intervention designs.
(3) Personalization of interventions: Exploring adaptive MBIs that cater to individual characteristics such as age, baseline psychological status, and music preference.
(4) Expansion into nonclinical contexts: Conducting further research to determine the effective implementation of MBIs in everyday settings, such as workplaces and educational institutions, to improve general well-being.
(5) Clinical integration: Findings suggest that MBIs—especially music therapy and music listening—hold promise for enhancing emotional well-being in clinical contexts. Future studies should explore how to effectively integrate these approaches into medical and psychological care, such as cancer treatment, cardiac rehabilitation, or mental health support. More research is also needed to determine optimal delivery formats, timing, and patient-tailored adjustments in clinical settings.

## 5 Conclusion

This study systematically evaluated the impact of MBIs (i.e., music therapy, training, and listening) on SWB in clinical and nonclinical populations. The results indicated that MBIs can effectively enhance SWB; however, the effects are significantly influenced by the type of intervention and characteristics of the population.

In clinical populations, music training yielded the most substantial benefits. Its structured and interactive nature may support emotional expression and foster social connections, which are particularly valuable in clinical contexts. Music listening also showed significant positive effects, possibly by engaging brain networks related to reward and emotion regulation. In comparison, while music therapy was effective, its effects were relatively weaker compared with those of the two other interventions. In contrast, a different trend was noted in the effects of MBIs in nonclinical populations, with music therapy exhibiting the most significant and positive effects. This finding may be attributed to its personalized design and interactive experience, thus effectively catering to the needs of nonclinical populations in managing daily emotions, including psychological needs. However, music training and listening exhibited no significant effects in nonclinical populations potentially due to relatively lower levels of emotional needs, insufficient interaction in the interventions, or a mismatch between training difficulty and their abilities.

The study further highlights that the long-term benefits of MBIs may depend on factors such as frequency, duration, and the presence of social reinforcement (e.g., group settings). Future research should explore how to tailor intervention types and delivery formats to specific populations, and investigate how participant characteristics (e.g., age, gender, psychological traits) influence outcomes. To improve replicability and comparability, the development of standardized intervention protocols is necessary (e.g., specifying duration, music type, and delivery mode). At the same time, incorporating flexible components that allow for personalization based on individual needs and contexts may help maximize intervention effectiveness. Interdisciplinary collaboration among psychology, neuroscience, and music therapy is recommended to better understand the mechanisms and optimize the design of MBIs for sustained well-being improvements.

## Data Availability

The original contributions presented in the study are included in the article/Supplementary material, further inquiries can be directed to the corresponding author.
