# Supplementary material for: Impact of music-based interventions on subjective well-being: a meta-analysis of listening, training, and therapy in clinical and nonclinical populations
Source: Front Psychol. 2025 Jul 9;16:1608508. doi: 10.3389/fpsyg.2025.1608508 (PMC12285531; doi:10.3389/fpsyg.2025.1608508)
Supplement: Supplementary file 4 [file Presentation_1.pdf]

Supplementary Material

Web of Science Search String：

TS=("Subjective Well-Being" OR "Emotional Well-Being" OR "Life Satisfaction" OR "Happiness" OR "Positive Affect") AND TS=("Music Intervention" OR "Music Therapy" OR "Music Training" OR "Music Listening" OR "Music Performance" OR "Music-based Intervention*") AND TS=("Adult*" OR "Patient*" OR "Clinical Population*" OR "Nonclinical" OR "Healthy Adult*" OR "Mental Health")

Filters： English; Article; Humans

Scopus Search String:

TITLE-ABS-KEY("Subjective Well-Being" OR "Emotional Well-Being" OR "Life Satisfaction" OR "Happiness" OR "Positive Affect") AND TITLE-ABS-KEY("Music Intervention" OR "Music Therapy" OR "Music Training" OR "Music Listening" OR "Music Performance" OR "Music-based Intervention*") AND TITLE-ABS-KEY("Adult*" OR "Patient*" OR "Clinical Population*" OR "Nonclinical" OR "Healthy Adult*" OR "Mental Health")

Filters： English; Article; Humans

PubMed Search String:

(("Subjective Well-Being"[Title/Abstract] OR "Emotional Well-Being"[Title/Abstract] OR "Life Satisfaction"[Title/Abstract] OR "Happiness"[Title/Abstract] OR "Positive Affect"[Title/Abstract]) AND ("Music Intervention"[Title/Abstract] OR "Music Therapy"[MeSH Terms] OR "Music Listening"[Title/Abstract] OR "Music Training"[Title/Abstract] OR "Music Performance"[Title/Abstract] OR "Music-based Intervention*"[Title/Abstract]) AND ("Adult"[MeSH Terms] OR "Adult*"[Title/Abstract] OR "Patient*"[Title/Abstract] OR "Clinical Population"[Title/Abstract] OR "Healthy Adults"[Title/Abstract] OR "Mental Health"[Title/Abstract]))

Filters： Humans; English; Journal Article
