# Supplementary figures and images for: Impact of music-based interventions on subjective well-being: a meta-analysis of listening, training, and therapy in clinical and nonclinical populations
Source: Front Psychol. 2025 Jul 9;16:1608508. doi: 10.3389/fpsyg.2025.1608508 (PMC12285531; doi:10.3389/fpsyg.2025.1608508)

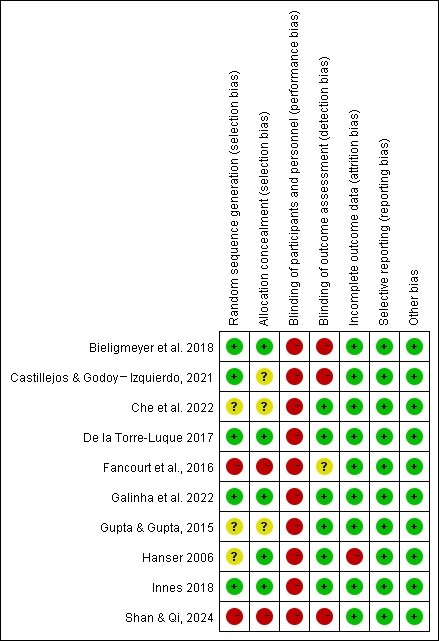

Supplement: Supplementary file 5 [file Image_1.jpeg]

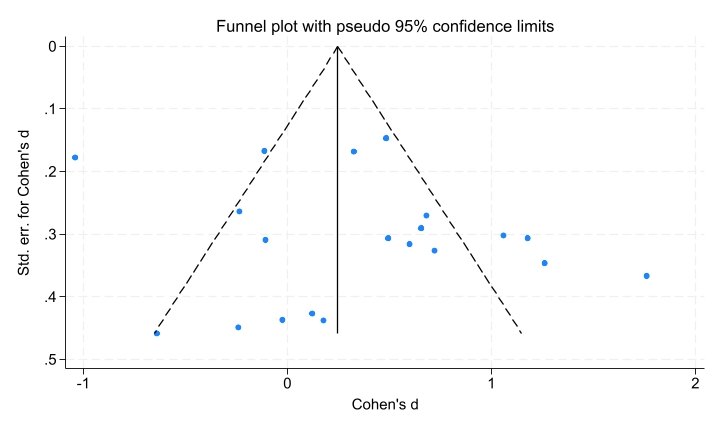

Supplement: Supplementary file 6 [file Image_2.jpeg]
